# A Case of Amaurosis, Induced by Terror

**Published:** 1819-06

**Authors:** Richard Philip Jones

**Affiliations:** Member of the Royal College of Surgeons.


*For the London Medical and Physical Journal.*

### *A Case of Amaurosis, induced by Terror;* by RICHARD PHILIP JONES, Esq. Member of the Royal College of Surgeons.

THE following case will serve to illustrate the influence of mental emotions in the production of disease. I am well aware it can afford no novelty to the generality of your readers; but I am confident, that some of the younger members of the profession (to whom I beg to offer it) will receive the communication with much satisfaction. This case occurred in a populous county in England, well supplied with medical practitioners; but, from the cause being novel in their history of disease, they were led too prematurely to give a hopeless decision. This rash judgment induced the unhappy patient to try the effect of *witchcraft*; and, accordingly, those marvellous pretenders exorcised, charmed, &c. and, in short, proceeded to the full extent of their incantations. At this period, when the patient's hope to regain his sight was reduced to the last ebb, I casually became a visitor in the neighbourhood; and, hearing of the circumstances, requested that I might see him. He had evidently recent amaurosis: But what a melancholy picture of debility he presented! His tongue was covered with a thick crust; his pulse weak and feeble; his speech very plaintive and faultering; and he suffered excessive nervous irritation. These symptoms had been much aggravated by the decision of the surgeons, &c.

About seven months previous to this time, he was attacked by some highway-robbers in the night, who, without inflicting personal injury at the time, left him in a state of half insensibility, proceeding from the terror he was thrown into. About a week after this occurrence, he found a considerable diminution of his sight, which gradually became worse; and the barren results of his medical consultations only urged the contemplation of each day to add greater poignancy to his already too-irritable disposition. I ordered him an emetic, with directions that it should be repeated regularly, followed by purgatives, with occasional doses of calomel conjoined with the tartarized antimony. He was also advised to be electrified three times a-week; to pay due observance to his diet; keep his mind tranquil; and to use regular and gentle exercise. The peculiar results which marked the commencement of this treatment produced such sanguine hopes of ultimate success, that he assiduously persisted in those means for about three months, when a complete cure was effected. It was truly interesting to mark the progressive symptoms of amendment: in proportion to the degree in which the excessive irritability was subdued, he enjoyed returning vision.

*Suffolk-street, London; March* 17, 1819.

